# *Elaeagnus angustifolia* L. Polysaccharide Alleviates High-Fat High-Fructose Diet (HFFD)-Induced Cognitive Impairment by Modulating the Gut-Liver-Brain Axis

**DOI:** 10.3390/foods15101794

**Published:** 2026-05-19

**Authors:** Bibinuer Yaermaimaiti, Shihua Huang, Hulalai Ayideng, Nuerxiayier Nazhaer, Naweire Yasen, Huiying Jing, Buweizuohere Tayier, Aiziguli Mulati

**Affiliations:** College of Food Science and Pharmacy, Xinjiang Agricultural University, Urumqi 830052, China; 15099561635@163.com (B.Y.); 15310666887@163.com (S.H.); 13209012831@163.com (H.A.); n17881217821@126.com (N.N.); 17799619206@163.com (N.Y.); wsxj7697@163.com (H.J.); 17799134855@163.com (B.T.)

**Keywords:** high-fat high-fructose diet (HFFD), *Elaeagnus angustifolia* polysaccharide, gut microbiota, short-chain fatty acids (SCFAs), neuroinflammation, gut–liver–brain axis, cognitive impairment

## Abstract

Cognitive impairment induced by a high-fat high-fructose diet (HFFD) is associated with gut–liver–brain axis dysfunction, yet whether polysaccharide intervention can modulate this axis to achieve cognitive rescue remains unexplored. This study investigated whether *Elaeagnus angustifolia* polysaccharide (EAP) is associated with protection against HFFD-induced cognitive decline by modulating this axis. Male C57BL/6J mice (*n* = 15/group) received Control, HFFD, HFFD + LEAP (300 mg/kg/day EAP), or HFFD + HEAP (800 mg/kg/day EAP) for 14 weeks. HEAP improved spatial memory, reducing escape latency by 31.2% on day 5 (*p* < 0.01). Multi-omics and histopathological analyses revealed that EAP was dose-dependently associated with restructuring of the gut microbiota, expanding Muribaculaceae and other SCFA-producers while suppressing pathobionts, thereby reversing the Firmicutes/Bacteroidota ratio from 1.71 to 0.94 (*p* < 0.01). Elevated cecal, hepatic, and cerebral acetate, propionate, and butyrate (*p* < 0.01) were associated with improved intestinal barrier integrity, attenuated systemic LPS translocation, and reduced hepatic inflammation and changes consistent with normalization toward control levels of PPARα/γ signaling. These peripheral improvements were accompanied by changes in the hippocampus, where EAP suppressed IBA-1 microglial activation (from 4.5-fold to 2.1-fold of control, *p* < 0.01) and IL-6/TNF-α signaling, changes in neurotransmitter balance (Glu, 5-HT, DA), and preserved postsynaptic density ultrastructure and PSD-95 expression (*p* < 0.01). These findings support a role for EAP in modulating the gut–liver–brain axis and may help prevent diet-related cognitive impairment, supporting its development as a microbiome-targeted functional food ingredient.

## 1. Introduction

Obesity, a disease characterized by the excessive accumulation of body fat, has evolved into a global epidemic and imposed a substantial economic burden on healthcare systems worldwide [1,2]. According to age-standardized estimates, the global obesity rate among adults was projected to rise from 14.5% in 2021 to 24.8% by 2050 [3]. The key drivers of the obesity epidemic included high-fat and high-fructose diets (HFFD), reduced physical activity, psychosocial stress, and sleep disturbances [4]. Individuals with obesity were at a markedly elevated risk of developing type 2 diabetes, hypertension, dyslipidemia, certain types of cancer, and musculoskeletal disorders [5,6].

HFFD consumption disrupted intestinal barrier integrity by downregulating tight junction proteins, including zonula occludens-1 and occludin. It also altered gut microbiota composition. These changes facilitated systemic lipopolysaccharide (LPS) translocation and metabolic endotoxemia [7,8]. Circulating LPS activated hepatic Toll-like receptor 4 and nuclear factor-κB (NF-κB) signaling. This triggered hepatic inflammation and steatosis, and elevated circulating free fatty acids and pro-inflammatory cytokines, such as tumor necrosis factor-α (TNF-α) and interleukin-6 (IL-6) [9,10]. These mediators compromised blood–brain barrier integrity. They activated microglia and astrocytes, leading to neuroinflammation. Ultimately, synaptic plasticity and cognitive function were impaired [11,12]. Consistent with these mechanisms, an 8-week HFFD regimen induced hippocampal atrophy, reduced synaptic density, and decreased brain-derived neurotrophic factor levels in animal models [13,14]. Concurrently, dysbiosis-driven alterations in bile acid metabolism amplified inflammatory signaling along the gut–liver–brain axis [15,16]. In this context, the application of functional bioactive compounds derived from specialty fruits has gained attention as a promising nutritional strategy to counteract HFFD-related metabolic disturbances.

*Elaeagnus angustifolia* L., commonly referred to as Russian olive or wild olive, is a deciduous shrub or small tree belonging to the Elaeagnaceae family. It is widely distributed across Asia, Europe, and North America [17]. *Elaeagnus angustifolia* polysaccharide (EAP) possesses antioxidant, anti-inflammatory, and metabolic-regulatory properties [18]. In diabetic models, EAP reduced fasting glucose and serum lipids [19]. In vitro, EAP exhibited potent free radical-scavenging capacity and inhibited α-glucosidase activity, indicating its dual potential to alleviate oxidative stress and regulate postprandial glucose [20]. Clinically, EAP improved lipid profiles and vascular function in menopausal women [21]. These findings indicate that EAP holds the potential to mitigate metabolic disorders.

Although EAP has been demonstrated to confer beneficial effects on metabolic disorders such as hyperglycemia and dyslipidemia, its potential to ameliorate HFFD-induced cognitive impairment remains unexplored. Moreover, no previous study has integrated multi-omics profiling within a gut–liver–brain axis framework to mechanistically link EAP-induced gut microbial changes with neuroprotection against diet-related cognitive decline. Therefore, the present study aimed to (i) evaluate the neuroprotective efficacy of EAP against HFFD-induced cognitive decline; (ii) elucidate the underlying mechanisms mediated through the gut–liver–brain axis; and (iii) investigate the relationship between diet-induced metabolic dysregulation and cognitive deficits. Together, these findings provide a mechanistic rationale for developing EAP as a functional food ingredient that may help prevent or mitigate diet-related cognitive impairment.

## 2. Materials and Methods

### 2.1. Animals and Experimental Design

**Figure 1:**
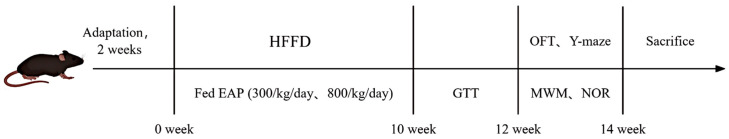
Experimental schedule of Elaeagnus angustifolia polysaccharide (EAP) treatment.

An in vivo mouse model of HFFD-induced metabolic dysfunction was established. Male C57BL/6J mice (8 weeks old) were obtained from Xinjiang Medical University (Urumqi, Xinjiang, China). Animals were randomly assigned to cages (five per cage) under controlled conditions (22 ± 2 °C, 50 ± 15% relative humidity, 12 h light/dark cycle) with free access to standard chow and water. Following a 2-week acclimation, mice were allocated to four dietary groups: (1) Control: AIN-93M standard diet, daily gavage with vehicle (pure water); (2) HFFD: high-fat diet (45% kcal from fat), daily gavage with vehicle (pure water); (3) HFFD + LEAP: HFFD plus 300 mg kg^−1^ day^−1^ EAP by gavage; and (4) HFFD + HEAP: HFFD plus 800 mg kg^−1^ day^−1^ EAP by gavage. These doses were selected based on preliminary dose-ranging studies in our laboratory, which indicated that 300 mg kg^−1^ day^−1^ exerted moderate metabolic effects, whereas 800 mg kg^−1^ day^−1^ provided optimal efficacy in improving glucose tolerance and lipid profiles without observable toxicity. Over a 14-week intervention period, gavage was administered once daily, and doses were recalibrated weekly according to individual body weight (Figure 1).

Every 3 days, mice were weighed, and ad libitum food and water consumption was recorded; the average daily energy intake (kcal/day/mouse) was calculated as follows: daily food intake (g/day) × energy density of diet (kcal/g). The food efficiency ratio (FER) was calculated for the entire 14-week experimental period as: [total body weight gain (g)/total energy intake (kcal)] × 100. All procedures were approved by the Animal Ethics Committee of Xinjiang Agricultural University (2025003) and conducted in accordance with the Guidelines for the Administration of Laboratory Animals issued by the State Committee of Science and Technology of the People’s Republic of China. All relevant ethical regulations regarding animal experimentation were strictly followed.

### 2.2. Glucose Tolerance Test (GTT), Sample Collection and Serum Analyses

At the end of the 14-week experimental period, mice were fasted for 12 h with free access to water. Following the fasting period, mice were anesthetized via inhalation of isoflurane (3% for induction, 1.5% for maintenance). Blood samples were collected from the retro-orbital sinus of mice under deep isoflurane anesthesia. Following blood collection, animals were humanely euthanized by cervical dislocation. The blood samples were allowed to clot at room temperature for 30 min and then centrifuged at 3000× *g* for 10 min at 4 °C to obtain serum. The brain, liver, and colon were rapidly excised. Cecal contents were aseptically collected into sterile tubes. All tissues were rinsed with ice-cold phosphate-buffered saline (PBS), blotted dry, and processed accordingly. For biochemical and molecular analyses, tissues were snap-frozen in liquid nitrogen and stored at −80 °C; for histopathological examination, tissues were fixed in 4% paraformaldehyde for 24 h at 4 °C.

Serum biochemical analyses. Serum obtained at the 14-week endpoint was used for the determination of total cholesterol (TC), triglyceride (TG), high-density lipoprotein cholesterol (HDL-C), and low-density lipoprotein cholesterol (LDL-C) by enzymatic colorimetric kits, and for the determination of insulin and LPS by ELISA kits (all from Xinle Bio, Shanghai, China). Homeostatic Model Assessment for Insulin Resistance (HOMA-IR) was calculated as: (fasting glucose concentration (mmol/L) × fasting insulin concentration (mIU/L))/22.5.

A GTT was performed at week 10 of the experimental period, separate from the terminal collection, according to established protocols [22]. Following a 12 h fast, mice received glucose (2 g/kg body weight) via oral gavage. Blood glucose concentrations were determined from the tail vein at 0, 30, 60, 90, and 120 min using a Sannuo glucometer(Sinocare Inc., Changsha, China). The area under the curve (AUC) for the GTT was computed using the trapezoidal rule.

### 2.3. Behavioral Tests

Extensive literature has demonstrated that prolonged HFFD feeding erodes learning and memory capacities in mice, culminating in overt cognitive dysfunction [23,24]. To assess behavioral alterations, open-field (OFT), Y-maze and novel-object recognition (NOR) tests were performed as previously described, followed by the Morris water maze (MWM) [25,26]. All behavioral tests were automatically captured and analyzed with SuperMaze video-tracking software (version 2.0, Xinruan Information Technology, Shanghai, China), with the exception of the open-field test (OFT), which was recorded and analyzed using Ethovision XT 14 software (Noldus Information Technology, Wageningen, The Netherlands). Essential procedural details for each test are outlined below.

The Y-maze test: Spontaneous alternation in the Y-maze has been shown to be one of the most sensitive and reproducible indices for assessing spatial working memory [27]. The apparatus consisted of three identical arms (35 × 5 × 15 cm) arranged at 120° to each other. The mouse was placed at the center of the maze and allowed to explore freely for 6 min. Entries into each arm were recorded, and consecutive visits to three different arms were defined as a single alternation. The percentage of alternations was calculated using the following formula: [the number of alternations/(total arm entries − 2)] × 100.

MWM: The MWM was widely used to evaluate spatial learning and memory [28]. The water maze apparatus comprised a circular pool (1.5 m diameter, 35 cm height) filled to a depth of 30 cm with 25 ± 1 °C water (XR-XM101, Shanghai Xinruan Information Technology Co., Ltd., Shanghai, China). The pool was equally divided into four quadrants (quadrants 1–4), and a transparent platform was hidden 1 cm below the water surface. During the acquisition phase, mice received four training trials per day for five consecutive days, with a maximum escape latency of 60 s per trial and an inter-trial interval of 15 min. If a mouse failed to locate the platform within the allotted time, it was gently guided to the platform and allowed to remain there for 15 s. On day 6, a probe trial was conducted by removing the platform and allowing the mouse to swim freely for 60 s. All trials were performed by an experimenter blinded to group allocation. Latency to reach the platform during acquisition, target-quadrant dwell time, and platform-crossing frequency during the probe trial were recorded as indices of spatial learning and memory.

NORT: The NORT was employed to evaluate non-spatial declarative memory [29]. After 6 min of free exploration in an empty arena (habituation), mice proceeded to the training phase. In the training phase, each mouse was placed in the arena for 6 min, now containing two identical, unscented objects. Following a 1 h retention interval, the 6 min test trial was performed: one familiar object remained, while the other was replaced with a novel item. Exploration was scored whenever the animal’s nose approached within a 2 cm radius of the object.

OFT: The OFT was conducted to assess open-field habituation and center exploration, which provide rapid, sensitive markers of hippocampal–prefrontal cognitive dysfunction [30]. Animals were gently placed in the center of a white, 50 cm × 50 cm × 40 cm open-field arena illuminated at 100 lux and allowed to explore for 5 min. Total distance and percentage time in the 25 × 25 cm central zone was automatically recorded and analyzed by Ethovision XT 14 software; the white arena was cleaned with 70% ethanol between trials.

### 2.4. Hematoxylin and Eosin (H&E) and Immunohistochemical Staining

H&E staining and immunohistochemistry (IHC) were performed according to established protocols [31]. Briefly, following euthanasia, colon, liver, and brain tissues were promptly collected and fixed in 4% paraformaldehyde (*v*/*v*) for 24 h, followed by paraffin embedding. Serial 4 µm sections were prepared for H&E staining and IHC. For IHC, deparaffinized and rehydrated sections underwent heat-induced antigen retrieval: sodium citrate buffer (10 mM, pH 6.0) was employed for IBA1, PPARα, and PPARγ, whereas EDTA buffer (1 mM, pH 9.0) was applied for MUC-2 and Claudin-1. Endogenous peroxidase activity was suppressed with 3% H_2_O_2_ for 10 min. Non-specific binding was prevented with 5% bovine serum albumin (BSA) in phosphate-buffered saline (PBS) for 1 h at room temperature. Sections were subsequently incubated overnight at 4 °C with the following primary antibodies diluted in PBS containing 1% BSA: rabbit anti-MUC-2 (1:200; ab90007, Abcam, Cambridge, UK), rabbit anti-Claudin-1 (1:100; 51-9000, Invitrogen, Waltham, MA, USA), rabbit anti-IBA1 (1:500; 019-19741, Fujifilm Wako, Osaka, Japan), rabbit anti-PPARα (1:200; ab24509, Abcam, Cambridge, UK), and rabbit anti-PPARγ (1:150; ab209350, Abcam, Cambridge, UK). After rinsing with PBS, sections were incubated for 1 h at room temperature with horseradish peroxidase (HRP)-conjugated goat anti-rabbit IgG (H + L) (1:500; 111-035-003, Jackson ImmunoResearch, West Grove, PA, USA) diluted in PBS containing 1% BSA. A signal was generated using 3,3′-diaminobenzidine (DAB), and sections were counterstained with Mayer’s hematoxylin. Negative controls were generated by omitting the primary antibody and substituting it with PBS containing 1% BSA. All slides were visualized under an optical microscope (Olympus BX53, Tokyo, Japan) and evaluated with ImageJ software (version 1.8.0, National Institutes of Health, Bethesda, MD, USA).

### 2.5. Analysis of the Ultrastructure of the Synapse in the Hippocampus

Hippocampal ultrastructure was examined using transmission electron microscopy according to established protocols [32]. For ultrastructural analysis of the hippocampus, micro-dissected tissue slices (≤1 mm^3^) were immediately fixed in 2.5% glutaraldehyde and 2% paraformaldehyde in 0.1 M phosphate buffer (PB, pH 7.4) at 4 °C for 4 h. Following primary fixation, samples were rinsed three times with 0.1 M PB (pH 7.4) and then post-fixed in 1% osmium tetroxide in the same buffer at 4 °C for 2 h in the dark. After osmication, the samples were rinsed with distilled water and dehydrated through a graded ethanol series (30%, 50%, 70%, 90%, and 100%; 10 min each). Samples were then infiltrated with a mixture of LR-White resin and absolute ethanol (1:1) overnight at 4 °C, followed by two changes of pure resin (2 h and 1 h, respectively) at 25 °C. Polymerization was performed at 60 °C for 24 h. After trimming, 70 nm ultrathin sections were cut using a diamond knife (Diatome, Biel, Switzerland) on an ultramicrotome (Leica Microsystems, Wetzlar, Germany), collected on 200-mesh formvar-coated copper grids, and double-stained with 2% uranyl acetate (5 min) and lead citrate (3 min). Sections were examined under a JEM-1230 transmission electron microscope (JEOL, Tokyo, Japan) operated at 80 kV. Digital images were captured with a side-mounted BioScan Veleta CCD camera (EMSIS GmbH, Münster, Germany).

### 2.6. Real-Time Quantitative PCR

Quantitative real-time PCR analyses were conducted according to published protocols [33]. RNA extraction: Total RNA from hippocampus, liver and colon was isolated using the RNA-Quick Purification Kit (ES Science, Shanghai, China) and quantified with an ND-100 micro-spectrophotometer; samples were then diluted to equal concentrations. Reverse transcription: First-strand cDNA was synthesized with RT Easy^™^ II Mix (RT-01022, Foregene, Chengdu, China) according to the manufacturer’s instructions. Real-time quantitative PCR: RT-qPCR was performed on an Accurate 96-X4 system (DLAB, Beijing, China) using SYBR Green master mix (QP-01012, Foregene, Chengdu, China). Ct values were normalized against glyceraldehyde-3-phosphate dehydrogenase (GAPDH), and relative gene expression was calculated using the 2^(−ΔΔCt) method. Table 1 shows gene-specific mouse primers.

### 2.7. Western Blotting

Total protein was extracted from hippocampal tissues using ice-cold RIPA lysis buffer (Beyotime, Shanghai, China) supplemented with 1% phenylmethylsulfonyl fluoride (PMSF) and a protease/phosphatase inhibitor cocktail (Beyotime). Tissue homogenates were sonicated on ice (3 bursts of 5 s each, with 10 s intervals) and centrifuged at 12,000× *g* for 15 min at 4 °C. The supernatant was harvested, and protein concentration was quantified using a BCA protein assay kit (Beyotime Biotechnology, Shanghai, China) in accordance with the manufacturer’s instructions. Equal amounts of protein (40 µg per lane) were combined with 5× SDS loading buffer, boiled at 95 °C for 5 min, resolved by SDS-PAGE on 8–15% polyacrylamide gels (the percentage was chosen based on the molecular weight of the target protein), and transferred onto 0.22 µm polyvinylidene difluoride (PVDF) membranes (Millipore, Burlington, MA, USA) at 300 mA for 60–90 min at 4 °C. Following transfer, membranes were blocked with 5% (*w*/*v*) non-fat dry milk in Tris-buffered saline containing 0.1% Tween-20 (TBST) for 1.5 h at room temperature [34]. Membranes were subsequently incubated overnight at 4 °C with the following primary antibodies diluted in TBST containing 5% bovine serum albumin (BSA): rabbit anti-PSD-95 (1:1000; ab238135, Abcam, Cambridge, UK), rabbit anti-IBA1 (1:1000; ab178846, Abcam, Cambridge, UK), and mouse anti-β-actin (1:5000; A5316, Sigma-Aldrich, St. Louis, MO, USA). After three washes with TBST (10 min each), membranes were incubated with horseradish peroxidase (HRP)-conjugated secondary antibodies for 1 h at room temperature: HRP-conjugated goat anti-rabbit IgG (H + L) (1:5000; 111-035-003, Jackson ImmunoResearch, West Grove, PA, USA) for PSD-95 and IBA1, and HRP-conjugated goat anti-mouse IgG (H + L) (1:5000; 115-035-003, Jackson ImmunoResearch) for β-actin. Immunoreactive bands were detected using an enhanced chemiluminescence (ECL) reagent (P0018M, Beyotime, Shanghai, China) and captured with a ChemiDoc imaging system (Bio-Rad, Hercules, CA, USA). Band intensities were analyzed using AlphaEaseFC software(version 4.0, Alpha Innotech, San Jose, CA, USA), normalized to the corresponding β-actin signal, and presented as fold-change relative to the control group.

### 2.8. 16S rDNA Microbiome Sequencing

Colon-content DNA was extracted by a CTAB/SDS method; purity and concentration were checked by NanoDrop and Qubit. The V3–V4 region was amplified with barcoded primers 341F (5′-CCTAYGGGRBGCASCAG-3′) and 806R (5′-GGACTACNNGGGTATCTAAT-3′) using high-fidelity polymerase. Amplicons were visualized on 2% agarose, excised, and purified with an AxyPrep DNA Gel Recovery kit. Library construction employed the NEB Next Ultra DNA Library Prep kit; libraries were quality-controlled using an Agilent 2100 Bioanalyzer (Agilent Technologies, Santa Clara, CA, USA) and quantified with Qubit, then sequenced on an Illumina platform. Raw reads were merged, filtered, and chimeras removed with UCLUST in QIIME 1.8.0. Clean reads were clustered into operational taxonomic units (OTUs) at 97% identity using UCLUST and annotated against the Greengenes database for taxonomic assignment and downstream diversity analyses. The OTU-based clustering approach was selected because it is the standard method integrated into the QIIME 1.8.0 pipeline used in this study, and it ensures comparability with existing datasets and prior publications from our group that were generated under the same bioinformatic protocol.

### 2.9. Plasma Metabolomics

Plasma (100 µL) was mixed with ice-cold methanol/acetonitrile/water (2:2:1, *v*/*v*), vortexed, and centrifuged (14,000× *g*, 4 °C, 20 min). The supernatant was injected into an Agilent 1290 Infinity UHPLC (Agilent Technologies, Santa Clara, CA, USA) coupled to an AB SCIEX Triple TOF 6600 (AB SCIEX, Framingham, MA, USA). Chromatographic separation was achieved on an ACQUITY UPLC BEH Amide column (1.7 µm, 2.1 × 100 mm) at 25 °C with 0.5 mL min^−1^ flow. Gradient: 0–0.5 min 95% B (acetonitrile), 0.5–7 min 95 → 65% B, 7–8 min 65 → 40% B, 8–9 min 40% B, 9–9.1 min 40 → 95% B, 9.1–12 min 95% B; injection volume 2 µL. ESI parameters: gas1 60, gas 260, CUR 30, TEM 600 °C, ISVF ± 5500 V; MS scan *m*/*z* 60–1000 (0.20 s), MS/MS *m*/*z* 25–1000 (0.05 s). IDA acquired 10 candidate ions per cycle (CE 35 ± 15 eV, DP ± 60 V). QC samples were interspersed to monitor drift. Features were annotated against an in-house database (Shanghai Applied Protein Technology, Shanghai, China) with a mass error of <25 ppm. A total of 186 and 183 metabolites were identified in RP^+^ and RP^−^ modes, respectively, with an annotation confidence level ≥ 2 PCA. PCA and OPLS-DA were performed; differential metabolites were selected with VIP > 1 and *p* < 0.05.

### 2.10. Neurotransmitter Quantification by UPLC-MS/MS

A targeted LC-MS/MS workflow was established for quantitative profiling of 57 neurotransmitters and related metabolites in mouse brain tissue. After thawing, 50 ± 2.5 mg tissue was extracted with 500 µL ice-cold 70% methanol (*v*/*v*), vortexed 3 min, and centrifuged (12,000 rpm, 4 °C, 10 min). The supernatant (300 µL) was re-centrifuged after 30 min at −20 °C, and 200 µL was injected (2 µL) onto an ACQUITY UPLC HSS PFP column (100 × 2.1 mm, 1.8 µm) operated at 40 °C. Chromatographic separation was achieved with a 0–7 min linear gradient from 5 to 95% acetonitrile containing 0.1% formic acid at 0.35 mL min^−1^. A SCIEX QTRAP 6500+ mass spectrometer (SCIEX, Framingham, MA, USA) equipped with an ESI source (5500 V positive, –4500 V negative, 550 °C) acquired MRM transitions optimized for each analyte. Quantification was performed against 18-point external calibration curves (0.01–10,000 ng mL^−1^) using MultiQuant 3.0.3, with QC samples interspersed every 10 injections to monitor instrumental stability (CV < 20%). Data were normalized to tissue weight and expressed as ng g^−1^ wet weight.

### 2.11. Bile Acid Quantification via LC-MS/MS

Liver tissue (20 mg) was accurately weighed into 2 mL Eppendorf tubes, fortified with 2 µL of a mixed internal standard solution (10 µg mL^−1^), and homogenized in 248 µL ice-cold methanol/acetonitrile (2:8, *v*/*v*) using a ball mill (30 Hz, 2 min). Protein precipitation was accomplished via incubation at −20 °C for 10 min, followed by centrifugation (12,000 rpm, 4 °C, 10 min). The supernatant was further purified through a 96-well protein-precipitation plate (Ostro, Waters, Milford, MA, USA), and 3 µL of the final extract was injected onto a Waters ACQUITY HSS T3 C18 column (100 × 2.1 mm, 1.8 µm) (Waters, Milford, MA, USA) maintained at 40 °C. Chromatographic separation was performed at 0.35 mL min^−1^ with a linear gradient of 5–95% B over 16 min, where mobile phase A consisted of 0.01% acetic acid plus 5 mmol L^−1^ ammonium acetate in water and mobile phase B comprised 0.01% acetic acid in acetonitrile. Mass spectrometric detection was conducted on an AB SCIEX QTRAP 6500+ system operated in negative electrospray ionization mode (capillary voltage –4.5 kV; source temperature 550 °C; curtain gas 35 psi). Scheduled multiple-reaction monitoring (sMRM) transitions were optimized for each analyte and internal standard. Peak integration and quantification were performed with MultiQuant 3.0.3 against 15-point external calibration curves (0.1–4000 ng mL^−1^). Quality control samples were injected every 10 study samples to monitor instrument performance; the acceptance criterion was CV < 20%. Final concentrations were normalized to wet tissue weight and reported as ng g^−1^.

### 2.12. Integrated Multi-Omics Analysis

To systematically explore the host–microbiota interactions, we integrated 16S rDNA microbiome sequencing data with untargeted metabolomics datasets. Briefly, after obtaining the microbial composition (OTU table) and metabolite abundance matrix, we performed data normalization and log-transformation. Multi-omics integration was conducted using Spearman rank correlation and orthogonal partial least squares discriminant analysis (OPLS-DA) to identify key microbial taxa and metabolites associated with phenotypic changes. Co-inertia analysis (CIA) and Procrustes tests were applied to evaluate the overall congruence between microbial and metabolite profiles. Additionally, microbiome–metabolite correlation analysis (MMCA) was performed using the ”mixOmics” R package (v6.18.0) to construct correlation networks and identify significant microbe–metabolite pairs (|r| > 0.6, FDR-adjusted *p* < 0.05). The integrated results were visualized using Cytoscape (v3.9.1) and R packages ggplot2, pheatmap, and corrplot.

### 2.13. Data Analysis

One-way ANOVA was performed using SPSS 27.0 (IBM Corp., Armonk, NY, USA) to evaluate differences among group means. Prior to ANOVA, the normality of data distribution was examined using the Shapiro–Wilk test, and the homogeneity of variances was evaluated using Levene’s test. Tukey’s honestly significant difference (HSD) post hoc test was employed for pairwise comparisons when a significant main effect was identified. For repeated-measures data (body weight, GTT, and MWM acquisition), two-way repeated-measures ANOVA was conducted with time as the within-subjects factor and group as the between-subjects factor; the Greenhouse–Geisser correction was utilized when the assumption of sphericity was violated. Non-parametric data were analyzed using the Kruskal–Wallis test, followed by Dunn’s post hoc test with Bonferroni adjustment. The level of statistical significance was established at *p* < 0.05 for all tests. Data were presented as mean ± standard error of the mean (SEM) and graphed using OriginPro 2022 (OriginLab Corporation, Northampton, MA, USA).

## 3. Results

### 3.1. Effects of EAP on Body Weight, Glucose Homeostasis, and Serum Lipid Profiles in HFFD-Fed Mice

**Figure 2:**
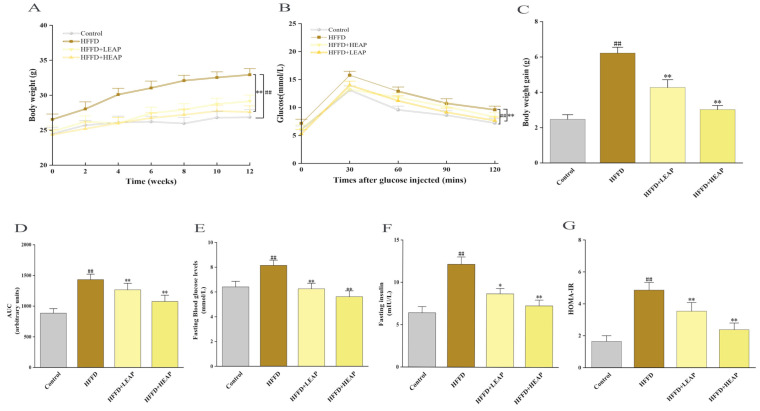
Effect of Elaeagnus angustifolia polysaccharide on body weight and glucose homeostasis in HFFD-induced obese mice. (**A**) Body weight; (**B**) GTT; (**C**) body weight gain; (**D**) AUC of GTT analysis; (**E**) fasting glucose concentration; (**F**) fasting insulin concentration; and (**G**) HOMA-IR. Data are presented as mean ± SEM, n = 15. ^##^ *p* < 0.01 versus control group, * *p* < 0.05, ** *p* < 0.01 versus HFFD group.

After the intervention period, the body weight of HFFD-fed mice was 23.0% higher than that of control mice. HEAP supplementation was associated with a reduction in this HFFD-induced net weight gain 49.0% (Figure 2A,C) (*p* < 0.01). Daily food and water intake did not differ among the HFFD-fed groups (Table 2). However, HEAP was associated with significantly lower food efficiency ratio and total energy intake in HFFD-fed mice (Table 2) (*p* < 0.01). Compared with the control group, HFFD feeding significantly increased serum levels of TC, TG, and LDL-C, while decreasing HDL-C (Table 2) (*p* < 0.01). HEAP treatment was associated with partial reversal of these changes, markedly lowering serum TG and TC levels and elevating HDL-C toward the levels observed in the control group (Table 2; *p* < 0.01).

EAP dose-dependently improved glucose tolerance, reducing the GTT-AUC by 11.5% (LEAP) and 24.9% (HEAP) relative to the HFFD group (Figure 2B,D) (*p* < 0.01). Fasting blood glucose was 23.2% and 31.0% lower in the HFFD + LEAP and HFFD + HEAP groups, respectively (Figure 2E) (*p* < 0.01), while fasting insulin declined by 28.6% and 40.1% (Figure 2F) (*p* < 0.01). The EAP treatment significantly inhibited (LEAP by 27.1% and HEAP by 51.0%) the HOMA-IR values in the HFFD-fed mice (Figure 2G) (*p* < 0.01).

### 3.2. Effects of EAP on Locomotor Activity, Working Memory, and Recognition Memory in HFFD-Fed Mice

**Figure 3:**
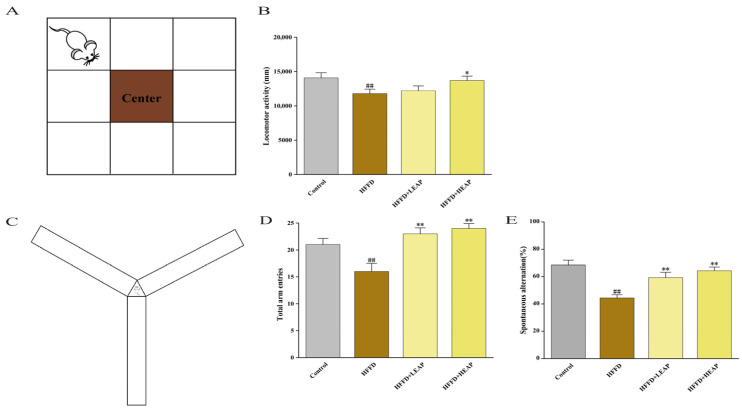
Effects of EAP on locomotor activity and working memory in HFFD-induced obese mice. (**A**) OFT diagram; (**B**) total distance; (**C**) Y-maze test diagram; (**D**) number of total arm entries; and (**E**) spontaneous alternation. Data are presented as mean ± SEM, n = 15. ^##^ *p* < 0.01 versus the control group, * *p* < 0.05, ** *p* < 0.01 versus the HFFD group.

The OFT apparatus consisted of a square arena with a central zone (Figure 3A). Compared with the control group, the HFFD group displayed a significant 18% decrease in spontaneous locomotor activity. Mice receiving EAP supplementation showed increased locomotor activity compared to the HFFD group (Figure 3B) (*p* < 0.05). The Y-maze apparatus consisted of three arms at equal angles (Figure 3C). Relative to the control group, HFFD feeding significantly decreased spontaneous alternation by 35.24% (Figure 3D,E) (*p* < 0.01). LEAP and HEAP were associated with increased spontaneous alternation by 33.74% and 45.09%, respectively, relative to the HFFD group (*p* < 0.05). Notably, neither dosage restored spontaneous alternation to control levels.

**Figure 4:**
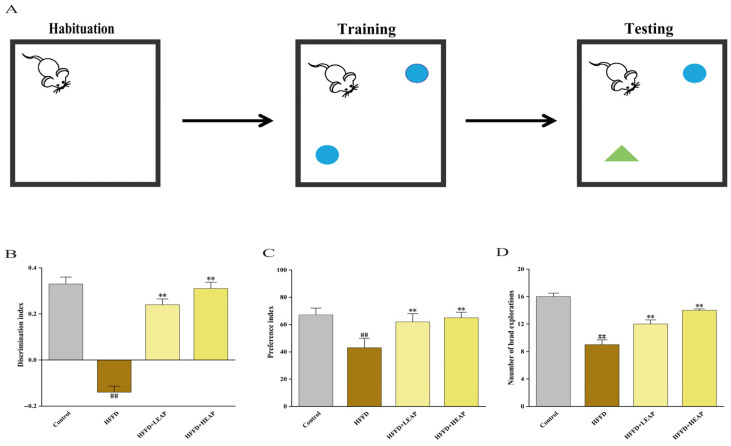
Effects of EAP on novel object recognition memory in HFFD-induced obese mice. (**A**) NOR test diagram; (**B**) discrimination index; (**C**) preference index; and (**D**) number of head explorations. Data are presented as mean ± SEM, n = 15. ^##^ *p* < 0.01 versus the control group, ** *p* < 0.01 versus the HFFD group.

The test consisted of three phases: habituation, training with two identical objects, and testing with one familiar and one novel object (Figure 4A). The HFFD group exhibited a discrimination index of −0.14, significantly lower than that of the control group (Figure 4B) (*p* < 0.01). The preference index decreased by 35.82% and head-exploration counts decreased by 43.75% in the HFFD group compared with the control group. LEAP and HEAP increased the preference index by 44.2% and 51.2%, respectively, and increased head-exploration counts by 33.3% and 55.6%, respectively, compared to the HFFD group (Figure 4C,D) (*p* < 0.01).

**Figure 5:**
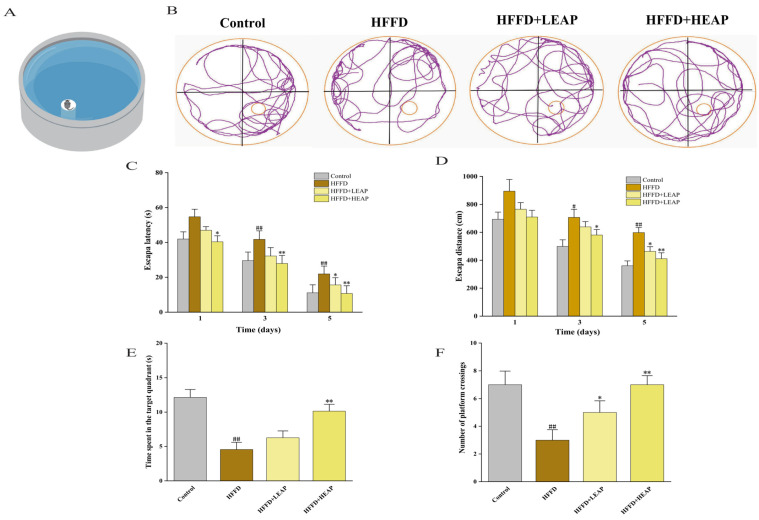
Effects of EAP on spatial learning and memory in HFFD-induced obese mice. (**A**) MWM test diagram; (**B**) representative swimming trajectories during the probe trial; (**C**) escape latency; (**D**) escape distance; (**E**) time spent in target quadrant; and (**F**) number of platform crossings. Data are presented as mean ± SEM, n = 15. ^#^ *p* <0.05, ^##^ *p* < 0.01 versus the control group, * *p* < 0.05, ** *p* < 0.01 versus the HFFD group.

The Morris water maze consisted of a circular pool with a hidden platform (Figure 5A). Representative swimming trajectories are shown in Figure 5B. During the acquisition phase, escape latency was shorter in the LEAP group (by 9.5% on day 3 and 22.5% on day 5) and the HEAP group (by 17.8% on day 3 and 31.2% on day 5) compared with the HFFD group (Figure 5C) (*p* < 0.01). Similarly, escape distance was decreased by LEAP and HEAP on day 3 and day 5 compared to the HFFD group (Figure 5D) (*p* < 0.05). In the probe trial, EAP-treated mice showed an increased duration in the target quadrant and an increased frequency of platform crossings compared with the HFFD group (Figure 5E,F) (*p* < 0.01).

### 3.3. Effects of EAP on Hippocampal Histopathology and Postsynaptic Density Ultrastructure in HFFD-Fed Mice

**Figure 6:**
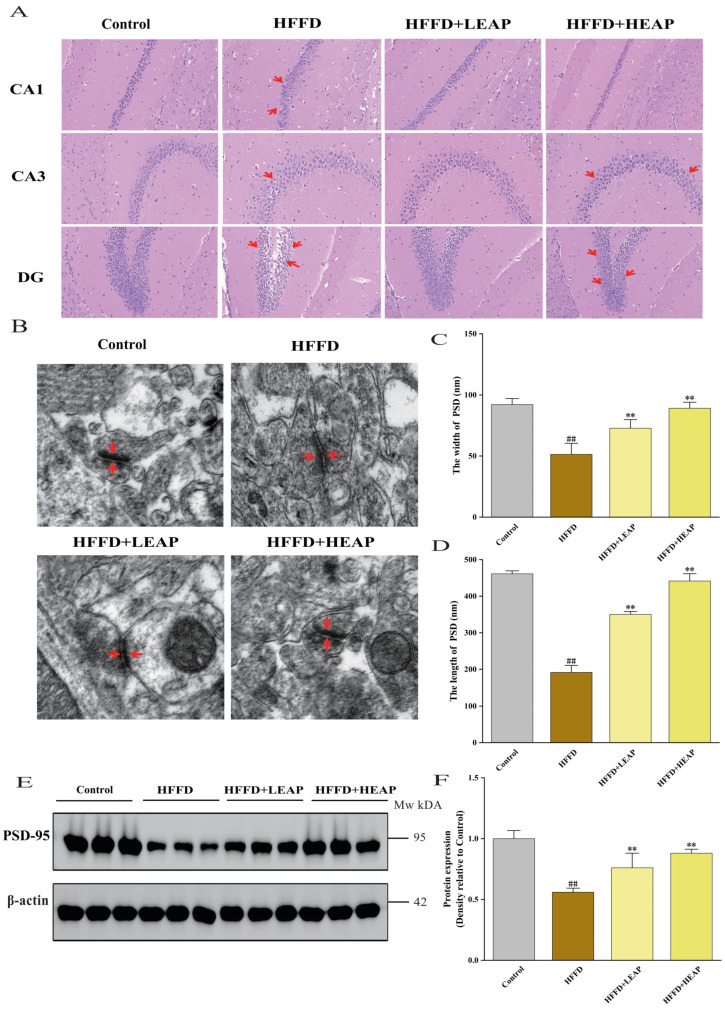
Effects of EAP on hippocampal and synaptic dysfunction in HFFD-induced obese mice. (**A**) Representative images of H&E staining of CA1/CA3/DG in the mouse hippocampus; (**B**) representative transmission electron micrographs of PSD-95; (**C**) width of PSD; (**D**) length of PSD; and (**E**) representative Western blots of PSD-95 in the mouse brain. β-actin was used as a loading control (n = 3 mice per group) and (**F**) densitometric analysis. Data are presented as mean ± SEM, n = 15. ## *p* < 0.01 versus the control group, ** *p* < 0.01 versus the HFFD group.

Hippocampal histopathology and postsynaptic density. H&E staining showed that pyramidal neurons in the cornu ammonis 3 (CA3) subfield of HFFD-fed mice were scattered compared to standard-diet controls. Neuronal arrangement in the CA1, CA3, and dentate gyrus (DG) was altered in HFFD-fed mice compared to controls; EAP supplementation changed neuronal morphology in these regions (Figure 6A). Postsynaptic density length and width decreased by 58.45% and 44.21%, respectively, in the HFFD group compared to the control group (Figure 6B–D) (*p* < 0.01). EAP supplementation was associated with increased postsynaptic density length and width compared to the HFFD group, although these parameters remained reduced relative to control levels (*p* < 0.01). PSD-95 expression was decreased in the HFFD group compared to the control group and was increased by EAP supplementation (Figure 6E,F) (*p* < 0.01).

### 3.4. Effects of EAP on Hippocampal Microglial Activation and Pro-Inflammatory Cytokine Expression in HFFD-Fed Mice

**Figure 7:**
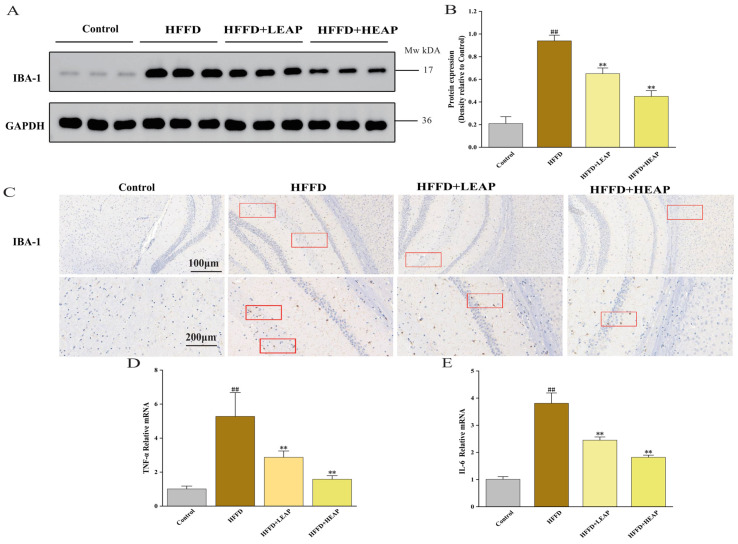
Effects of EAP on neuroinflammation in HFFD-induced obese mice. (**A**) Representative Western blots of IBA-1; (**B**) densitometric analysis; (**C**) representative Iba-1 immunoreactivity in the hippocampus; and (**D**,**E**) the mRNA levels of IL-6 and TNF-α. Data are presented as mean ± SEM, n = 15. ## *p* < 0.01 versus the control group, ** *p* < 0.01 versus the HFFD group.

IBA-1 expression was increased 4.5-fold in the HFFD group compared with the control group (*p* < 0.01). LEAP and HEAP reduced IBA-1 3.1-fold and 2.1-fold, respectively, compared to the HFFD group (Figure 7A,B) (*p* < 0.01). IBA-1-positive microglia in HFFD-fed mice showed hypertrophied somata with retracted processes; EAP supplementation changed microglial morphology (Figure 7C). IL-6 mRNA increased 3.8-fold, and TNF-α mRNA increased 5.2-fold in the HFFD group compared to the control group (*p* < 0.01). LEAP reduced these transcripts 2.4-fold (IL-6) and 2.8-fold (TNF-α), and HEAP decreased them 1.8-fold and 1.6-fold, compared to the HFFD group (Figure 7D,E) (*p* < 0.01).

### 3.5. Effects of EAP on Hepatic Histopathology, PPARα/γ Expression, and Hepatic Cytokine mRNA Levels in HFFD-Fed Mice

**Figure 8:**
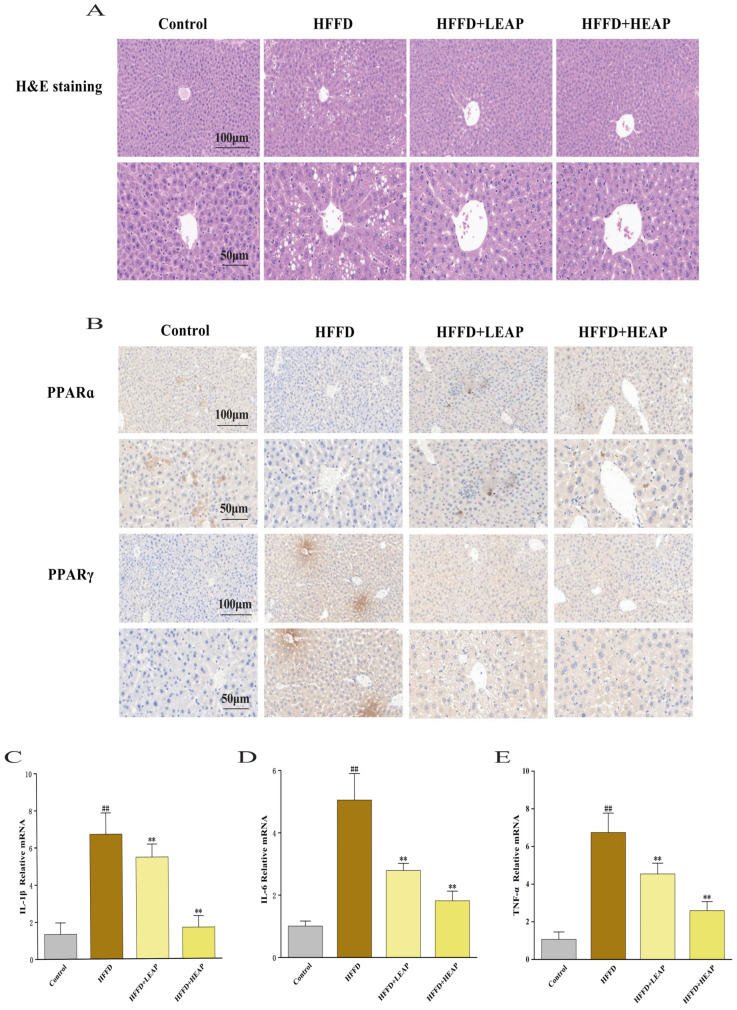
Effects of EAP on hepatic inflammation and PPARα/γ signaling in HFFD-induced obese mice. (**A**) Representative H&E-stained images of liver sections from each group; (**B**) representative PPARα/γ immunoreactivity in liver sections; (**C**–**E**) the mRNA levels of IL-1β, IL-6 and TNF-α. Data are presented as mean ± SEM, n = 15. ^##^ *p* < 0.01 versus the control group, ** *p* < 0.01 versus the HFFD group.

H&E staining showed lobular architecture with radially arranged hepatocytes in control livers. In contrast, HFFD livers showed lobular disarray, diffuse cytoplasmic lipid vacuoles, focal necrosis, and inflammatory infiltrate. EAP supplementation reduced lipid vacuoles and inflammatory infiltrate in HFFD-fed mice (Figure 8A). PPARα-positive granules were distributed in control livers, whereas PPARα immunoreactivity was decreased in HFFD-fed mice. LEAP and HEAP increased PPARα immunoreactivity compared to the HFFD group (Figure 8B). Conversely, PPARγ-positive cells were sparse in control livers; the number and intensity of PPARγ-positive cells were increased in HFFD-fed mice, predominantly around central veins. LEAP and HEAP decreased PPARγ-positive cell counts and staining intensity compared to the HFFD group (Figure 8B). At the mRNA level, IL-6, IL-1β, and TNF-α mRNA were increased 5.0-fold, 5.0-fold, and 6.3-fold, respectively, in the HFFD group compared with the control group (*p* < 0.01). LEAP was associated with a 2.8-fold (IL-6), 4.1-fold (IL-1β), and 4.2-fold (TNF-α) reduction of these transcripts, respectively, and HEAP was associated with a 1.8-fold, 1.3-fold, and 2.4-fold reduction, respectively, compared with the HFFD group (Figure 8C–E) (*p* < 0.01).

### 3.6. Effects of EAP on Short-Chain Fatty Acid Levels in Cecal Contents, Liver, and Brain of HFFD-Fed Mice

**Figure 9:**
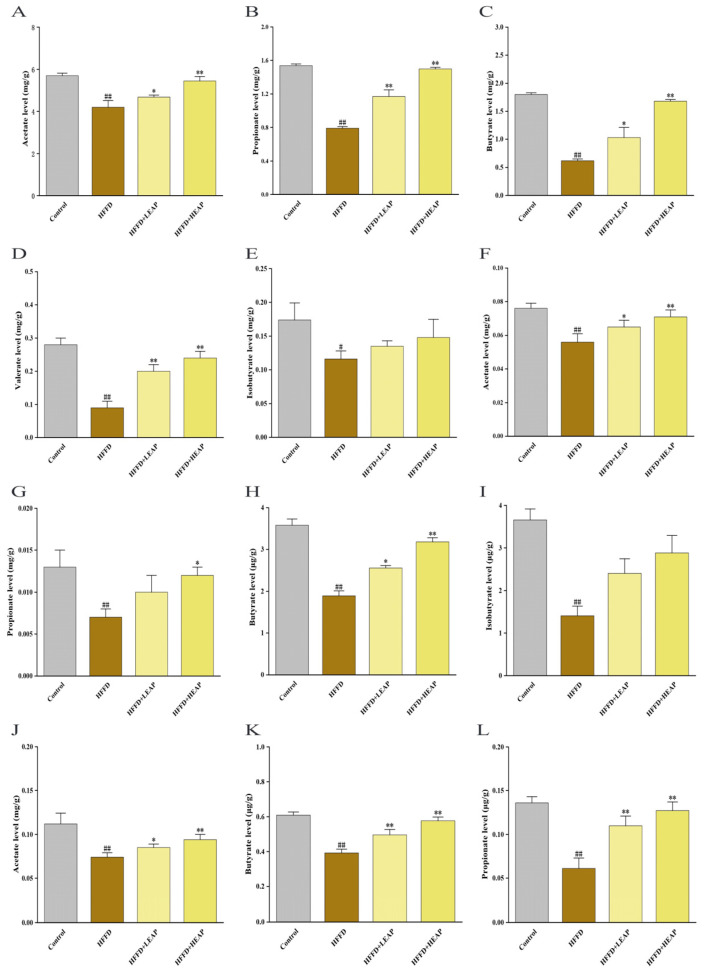
Effects of EAP on gut–liver–brain short-chain fatty acid metabolome in HFFD-induced obese mice. (**A**–**E**) Amounts of acetic, propionic, butyric, valeric, and isobutyric acids in cecal contents; (**F**–**I**) hepatic contents of acetic, propionic, butyric and isobutyric acids; (**J**–**L**) cerebral contents of acetic, propionic and butyric acids. Data are presented as mean ± SEM, n = 15. ^#^ *p* <0.05, ^##^ *p* < 0.01 versus the control group, * *p* < 0.05, ** *p* < 0.01 versus the HFFD group.

In the cecum, HFFD feeding was associated with decreased concentrations of acetic acid by 26%, propionic acid by 49%, butyric acid by 66%, and valeric acid by 68% compared with the control group (*p* < 0.01). LEAP elevated these SCFAs toward control levels (78%, 76%, 57%, and 71% of control values, respectively). Furthermore, HEAP elevated acetic and propionic acids compared with the HFFD group (*p* < 0.01). HFFD also lowered isobutyric acid by 33% compared with the control group (*p* < 0.05). LEAP and HEAP raised isobutyric acid by 22% and 30%, respectively, compared with the HFFD group, although neither differed from the control group (Figure 9A–E) (*p* > 0.05). In the liver, HFFD decreased acetic acid by 26.3%, propionic acid by 46.2%, and butyric acid by 47.2% compared with the control group. LEAP elevated these SCFAs toward control levels (85.5%, 76.9%, and 71.5% of control values, respectively). Similarly, HEAP increased these SCFAs compared with the HFFD group (Figure 9F,I) (*p* < 0.01). HFFD reduced hepatic isobutyric acid by 61.6%. LEAP and HEAP elevated isobutyrate toward control levels (65.7% and 76.0% of control, respectively); neither differed from the control group (*p* > 0.05). In the brain, HFFD reduced whole-brain acetic acid, propionic acid, and butyric acid levels by 34%, 55%, and 36%, respectively, compared with the control group. LEAP and HEAP elevated these metabolites compared with the HFFD group (Figure 9J–L) (*p* < 0.01).

### 3.7. Effects of EAP on Cerebral Neurotransmitter Levels and Hepatic Bile Acid Profiles in HFFD-Fed Mice

**Figure 10:**
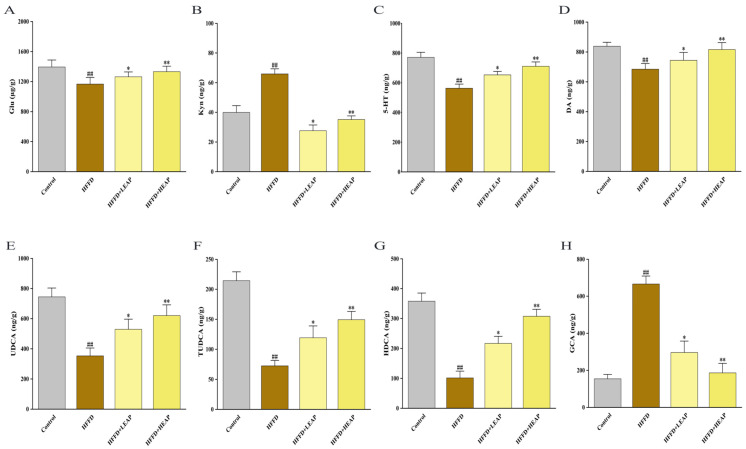
Effects of EAP on cerebral neurotransmitters and bile acids in HFFD-induced obese mice. (**A**–**D**) Cerebral levels of glutamate (Glu), kynurenine (Kyn), 5-hydroxytryptamine (5-HT) and dopamine (DA); and (**E**–**H**) hepatic levels of ursodeoxycholic acid (UDCA), tauroursodeoxycholic acid (TUDCA), hyodeoxycholic acid (HDCA) and glycocholic acid (GCA). Data are presented as mean ± SEM, n = 15. ^##^ *p* < 0.01 versus the control group, * *p* < 0.05, ** *p* < 0.01 versus the HFFD group.

Relative to the control group, HFFD feeding was associated with 16.3% lower cortical glutamate (Glu), 27.0% lower 5-hydroxytryptamine (5-HT), and 18.3% lower dopamine (DA), as well as 65.0% higher kynurenine (Kyn). LEAP partially reversed these changes, increasing Glu (8.3%), 5-HT (16.0%), and DA (8.7%), while decreasing Kyn (58.1%) relative to the HFFD group. HEAP exerted similar effects (Figure 10A–D) (*p* < 0.01).

HFFD decreased UDCA, TUDCA, and HDCA by 53%, 66%, and 72%, respectively, and increased GCA 4.3-fold compared to the control group. LEAP increased UDCA, TUDCA, and HDCA and decreased GCA compared to the HFFD group. HEAP increased UDCA, TUDCA, and HDCA, and decreased GCA compared to the HFFD group (Figure 10E–H) (*p* < 0.01).

### 3.8. Effects of EAP on Intestinal Barrier Integrity and Gut Microbiota Composition in HFFD-Fed Mice

**Figure 11:**
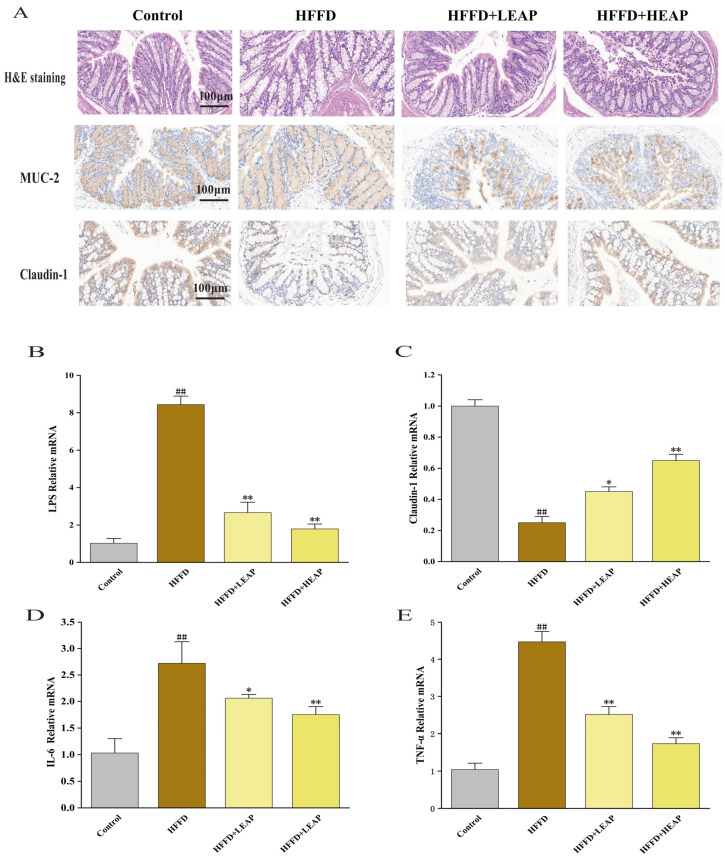
Effects of EAP on intestinal barrier dysfunction in HFFD-induced obese mice. (**A**) Representative H&E staining and immunoreactivity for MUC-2 and Claudin in mouse colonic mucosa; (**B**–**E**) the mRNA expression levels of LPS, Claudin-1, IL-6, and TNF-α. Data are presented as mean ± SEM, n = 15. ^##^ *p* < 0.01 versus the control group, * *p* < 0.05, ** *p* < 0.01 versus the HFFD group.

H&E staining revealed severe mucosal disruption in the proximal colon of HFFD-fed mice, including crypt distortion, irregular branching, and widespread goblet cell loss. EAP treatment was associated with improved crypt regularity (Figure 11A). Immunohistochemical analysis showed markedly reduced MUC-2 expression within colonic crypts of HFFD-fed mice, with discontinuous goblet-cell staining; EAP increased MUC-2 expression toward control levels. In parallel, HFFD-fed mice exhibited loss of Claudin-1 along the crypt epithelium, with disrupted and faint junctional staining. These alterations were less pronounced following EAP supplementation (Figure 11A). HFFD feeding was associated with increased ileal LPS (8.3-fold), IL-6 (2.6-fold), and TNF-α (4.3-fold) mRNA levels, and decreased Claudin-1 (to 25% of that of the control group). LEAP reduced these changes 2.6-fold, 2.0-fold, 2.4-fold, and to 45% of that of the control group, respectively; HEAP reduced them to 1.8-fold, 1.7-fold, 1.7-fold, and to 65% of that of the control group, respectively (Figure 11B–E) (*p* < 0.01).

**Figure 12:**
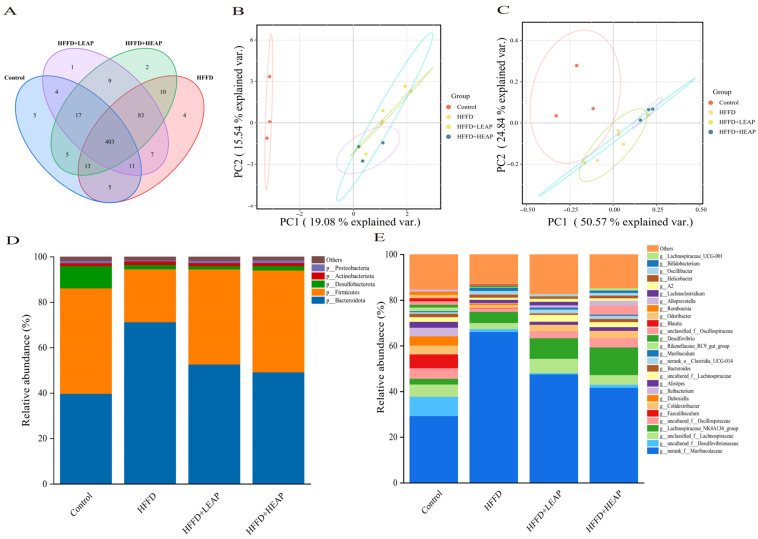
(**A**) Venn diagram illustrating the discrepancy of OTUs among the different treatment groups; (**B**,**C**) PCA and PCoA diagram; (**D**) relative abundance at the phylum level; and (**E**) relative abundance at the genus level. Data are presented as mean ± SEM, n = 15. versus the control group, versus the HFFD group.

We further investigated the effects of EAP on HFFD-induced gut microbiota dysbiosis in mice. The Venn diagram showed that the Control, HFFD, HFFD + LEAP, and HFFD + HEAP groups had 5, 4, 1, and 2 unique OTUs, respectively (Figure 12A). Principal coordinate analysis (Bray–Curtis distance) revealed a consistent diet-driven segregation of cecal microbiotas in both experiments. In the first trial (Figure 12B), PC1 explained 19.1% of the total variance and separated HFFD microbiotas from controls along the primary axis; HFFD + LEAP and HFFD + HEAP interventions occupied intermediate positions, with HFFD + HEAP falling closest to the control cloud. A second, independent cohort (Figure 12C) reproduced this pattern with even greater resolution: PC1 now captured 50.6% of the variance, yielding a tight control cluster that was clearly disjoint from the HFFD centroid, whereas HFFD + HEAP samples again clustered most proximally to controls, and HFFD + LEAP remained nearer to the HFFD group. Relative abundance analysis at the phylum level revealed that the gut microbiota in control mice was predominantly composed of Bacteroidota (55%) and Firmicutes (40%), collectively accounting for over 80% of the total microbial sequences. HFFD feeding was associated with altered phylum-level composition: higher Firmicutes (~60%) and lower Bacteroidota (35%), yielding an elevated Firmicutes/Bacteroidota (F/B) ratio (0.73 to 1.71). Both LEAP and HEAP were associated with shifts toward control levels, with HEAP showing Firmicutes and Bacteroidota at 45% and 48%, respectively, and an F/B ratio of 0.94 (Figure 12D). At the genus level, Muribaculaceae (Muribaculum) dominated the cecal microbiota of control mice, accounting for 80% of the relative abundance. HFFD feeding was associated with a lower proportion (20%), a 60-percentage point decrease. LEAP and HEAP were both associated with higher Muribaculum abundance (25% and 45%, respectively). These changes paralleled increased total cecal concentrations of acetate, propionate, and butyrate (78%, 76%, and 57% of control values, respectively). In contrast, the decrease in isobutyric acid—a marker of protein fermentation—remained evident, with levels 22–30% lower than those of the control (Figure 12E).

LEfSe analysis (LDA > 2.0) revealed markedly distinct bacterial signatures among the three experimental groups (Figure S1A,B). In HFFD-fed mice, 28 taxa were significantly enriched, nearly all of which were classified under the phylum Firmicutes (Figure S1A). The most prominent biomarkers included members of the families Lachnospiraceae and Oscillospiraceae. Additional HFFD-associated taxa encompassed Prevotellaceae, Tyzzerella, Incertae Sedis, Anaerococcus, Peptococcus, and Staphylococcus (Figure S1A), collectively indicating a Firmicutes-dominated dysbiotic state.

In contrast, the control group microbiota exhibited 31 discriminative features that were largely absent in HFFD-fed mice (Figure S1B). These biomarkers were predominantly affiliated with Bacteroidota, particularly Muribaculaceae and Bacteroidaceae. Firmicutes enriched in the control group comprised Christensenellaceae, Peptostreptococcaceae, Erysipelatoclostridiaceae, and several Clostridia lineages. Other control-associated biomarkers included Desulfovibrionaceae, Coriobacteriia, and RF39 (Figure S1B).

Notably, the HFFD + HEAP group exhibited a substantial reversal of the HFFD-induced biomarker profile: none of the Firmicutes OTUs enriched in the HFFD group remained significantly abundant in the HEAP group. Furthermore, the HEAP group acquired 27 out of the 31 control-discriminatory taxa, including all Muribaculaceae members, *Bacteroides vulgatus*, *Christensenella minuta*, *Romboutsia ilealis*, and Desulfovibrionaceae (Figure S1B). Together, these findings show that HEAP supplementation effectively reverses HFFD-induced gut microbiota dysbiosis and restores a microbiome structure rich in Bacteroidota and Christensenellaceae, which is consistent with a healthy microbial ecology. LEfSe revealed significant taxonomic divergence among the four groups (LDA > 0.7). HFFD-fed mice exhibited enrichment of potential pathobionts (*Desulfovibrio*, *Bilophila* and *Clostridium*; LDA > 1.1, blue bars) and a concomitant depletion of fiber-degrading commensals, including norank f_Muribaculaceae, Bifidobacterium and Roseburia (negative LDA scores, orange bars). Both LEAP and HEAP interventions reversed these diet-induced shifts in a dose-dependent manner, restoring short-chain fatty acid producers and re-establishing a health-associated microbiome profile (Figure S2).

## 4. Discussion

### 4.1. Interpretation of Key Findings

The present study demonstrates that a dose-dependent association between EAP and neuroprotection against HFFD-induced cognitive impairment. HEAP (800 mg/kg/day) was associated with greater cognitive improvements than LEAP (300 mg/kg/day), consistent with a microbial threshold for microbiome-mediated neuroprotection. The nonlinear dose response aligns with prior polysaccharide interventions [16,35]. However, our findings extend these observations by suggesting a specific microbial threshold: normalization of the Firmicutes/Bacteroidota ratio to 0.94 coincided with substantial increases in cerebral SCFA levels and was associated with synaptic integrity, although causal priority remains undetermined.

Differences in microbial composition between LEAP and HEAP may contribute to their differential cognitive outcomes. The incomplete increase in Muribaculaceae abundance by LEAP (25% vs. 45% under HEAP) coincided with lower cerebral SCFA levels, suggesting that Muribaculaceae abundance may need to reach a critical threshold to generate sufficient SCFAs for neuroimmune modulation. In contrast, the persistent deficit in isobutyric acid associated with both doses (22–30% below control, *p* > 0.05) suggested that EAP selectively targeted carbohydrate-fermenting pathways rather than protein fermentation. This prebiotic-like selectivity appeared distinct from the broad microbial stimulation reported for less refined fiber preparations [36,37,38].

From a translational perspective, the HEAP dose approximated a 6.5-fold increase over typical human dietary polysaccharide intake. This raises formulation challenges for clinical application. Encapsulation or colonic delivery systems may be necessary to achieve comparable microbial engagement without excessive bulk intake.

### 4.2. Mechanistic Insights: A Directional Gut–Liver–Brain Cascade

The concurrent improvement in gut barrier function, hepatic metabolism, and hippocampal synaptic integrity across EAP-treated mice suggested a directional gut–liver–brain cascade rather than independent compartmental effects. Although the cross-sectional design precludes definitive causal inference, the magnitude and coherence of multi-omics changes support a plausible sequential model warranting longitudinal validation.

At the intestinal level, EAP was associated with increased MUC-2 and Claudin-1 expression compared with control levels. This dual reinforcement of mucus and tight-junction barriers likely reduced paracellular LPS translocation. Circulating LPS declined under HEAP, consistent with attenuated ileal inflammatory signaling. These findings align with reports that metabolic endotoxemia drives neuroinflammation in diet-induced obesity [39]. However, the dose-dependent nature of barrier restoration in our study—partial under LEAP, robust under HEAP—suggests that a threshold level of mucosal integrity may be required to suppress systemic endotoxemia sufficiently for downstream neuroprotection. Direct permeability measurements would be needed to confirm this mechanism.

Hepatic metabolic reprogramming appeared to serve as the critical mediator between gut improvement and brain protection. EAP was associated with changes in PPARα/γ signaling coincident with SCFA elevation. This microbiota-dependent regulation contrasts with direct PPAR agonists such as fibrates and thiazolidinediones, which activate receptors independently of microbial metabolism [40]. This distinction may be clinically relevant: direct agonists induce systemic PPARγ activation with adipogenic side effects, whereas EAP was associated with enhanced hepatic lipid oxidation via PPARα without overt adipogenesis. This tissue selectivity supports the functional food paradigm, wherein endogenous metabolic crosstalk is leveraged rather than pharmacologically overridden. Whether PPAR normalization was a consequence of SCFA recovery, a parallel pathway, or both remains unresolved. Hepatocyte-specific PPAR knockout studies combined with EAP treatment would clarify this relationship.

In the hippocampus, SCFA elevation coincided with microglial quiescence and reduced pro-inflammatory cytokine expression. These observations resonated with demonstrations that SCFAs suppress LPS-induced microglial activation through FFAR2 signaling and HDAC inhibition [41]. Yet an apparent contradiction merits scrutiny: propionate was reported to exacerbate neuroinflammation in high-fat diet models [42]. We speculate that this discrepancy reflects context-dependent effects—propionate administered in isolation or at supraphysiological doses may trigger distinct immune responses compared with the balanced SCFA milieu (acetate, propionate, butyrate) restored by EAP. The incomplete normalization of IL-6 and TNF-α transcripts despite near-complete hippocampal SCFA recovery further implicated additional mechanisms. Direct polysaccharide bioactivity or bile acid–TGR5/FXR signaling may contribute, as EAP concurrently increased neuroprotective UDCA and TUDCA while reducing GCA accumulation [43].

The source of cerebral SCFAs remains unclear. The magnitude of hippocampal SCFA recovery under HEAP exceeded predictions from passive diffusion models [44]. Two competing hypotheses emerged: (i) active transport or metabolic conversion within the CNS, or (ii) peripheral SCFA-mediated suppression of systemic inflammation with secondary reduction of neuroinflammatory tone. Isotope-labeled SCFA tracing studies would distinguish these possibilities. Finally, the preferential elevation of carbohydrate-fermenting SCFAs over protein fermentation markers (isobutyric acid remained 22–30% below that of the control, *p* > 0.05) indicated that EAP functioned as a selective prebiotic substrate. This glycan-directed specificity for saccharolytic taxa (Muribaculaceae, Bacteroidaceae) may avoid the off-target expansion of proteolytic pathobionts reported with less selective fibers [45]. Such selectivity has important translational implications: EAP’s neuroprotective efficacy may depend on preserving a balanced fermentation profile rather than simply increasing total SCFA output.

### 4.3. Comparison with the Existing Literature: Agreement and Discrepancy

Our findings align with studies demonstrating that polysaccharide interventions preserve cognition through gut microbial remodeling. *Lycium barbarum* polysaccharide was associated with restored cognitive performance by enriching Bacteroidetes and elevating fecal acetate and butyrate [46]; *Astragalus membranaceus* polysaccharide normalized the Firmicutes/Bacteroidota ratio in diabetic mice [47]. EAP extended this paradigm by identifying a dose-dependent microbial threshold-F/B ratio of 0.94, below which cognitive rescue remained incomplete, and by demonstrating simultaneous elevations in SCFA levels across the cecal, hepatic, and cerebral compartments. Prior studies typically measured single-site SCFAs. However, not all microbiome-targeted interventions engaged SCFA-dependent mechanisms. *Bifidobacterium longum* improved stress-related cognition primarily through vagal afferent signaling rather than SCFA production [48]. This divergence likely reflects intervention-specific properties rather than mutually exclusive pathways: vagal signaling operates on rapid neural timescales, whereas SCFA-mediated effects involve slower transcriptional modifications. EAP’s glycan structure may favor saccharolytic fermentation, determining its preferred signaling route.

Our observation of hippocampal SCFA restoration also challenged the prevailing assumption that brain SCFAs derive primarily from passive BBB diffusion [44,49]. The magnitude of cerebral recovery under HEAP exceeded pharmacokinetic predictions, suggesting either active CNS transport or peripheral SCFA-mediated suppression of systemic inflammation with secondary neuroimmune benefits. Isotope-labeled tracing would distinguish these possibilities. Finally, prior studies have examined isolated gut–brain [50] or gut–liver [51] connections. Our integrated multi-omics approach captured simultaneous restoration across all three compartments, suggesting that effective neuroprotection may require coordinated multi-organ metabolic repair rather than isolated target modulation.

### 4.4. Limitations and Future Directions

Several limitations warrant consideration. First, the exclusive use of male mice restricted generalizability. HFFD-induced neuroinflammation exhibits distinct temporal dynamics and microbiome responses in females [52]. The observed dose-response relationship may therefore differ by sex. Second, the 14-week intervention captured early metabolic dysfunction but not the chronic neurodegenerative progression relevant to aging-related dementia. Third, the terminal endpoint design precluded establishing temporal precedence. The sequence of SCFA elevation relative to microglial suppression remains a critical step for causal inference.

Methodological constraints also merit acknowledgment. Intestinal permeability was inferred from histological and molecular markers rather than measured directly. Serum zonulin or FITC-dextran assays would strengthen barrier function claims in future work. Circulating LPS was quantified by ELISA without endotoxin bioactivity assessment by LAL assay. Additionally, the relative contributions of EAP’s direct bioactivity versus microbiota-mediated effects remain unclear. Germ-free mouse colonization or antibiotic depletion studies would clarify this distinction.

From a translational perspective, the human equivalent dose exceeds typical dietary polysaccharide intake. Encapsulation or colonic delivery systems may be necessary to achieve comparable microbial engagement. Pharmacokinetic studies in humans must address bioavailability, as oral EAP may undergo gastric degradation. EAP could be positioned as a targeted prebiotic for metabolic syndrome patients with early cognitive decline, particularly given its tissue-selective PPAR modulation. However, some nutritional interventions improve peripheral metabolism without cognitive benefit [53]. Future clinical trials should therefore prioritize cognitive endpoints, independently of metabolic changes, to establish EAP’s neuroprotective efficacy.

## 5. Conclusions

This study demonstrates that *Elaeagnus angustifolia* polysaccharide supplementation is associated with reversal of HFFD-induced cognitive impairment and coordinated changes across the gut–liver–brain axis. The key novel findings are threefold. First, EAP’s neuroprotective efficacy appears to extend beyond its previously documented metabolic benefits, with associated changes in hippocampal synaptic preservation, neurotransmitter balance, and spatial memory performance. Second, robust protection was associated with normalization of the Firmicutes/Bacteroidota ratio to approximately 0.94 and concomitant SCFA changes across cecal, hepatic, and cerebral compartments, suggesting a dose-dependent microbial threshold. Third, hippocampal SCFA changes coincided with microglial quiescence, reduced pro-inflammatory cytokine expression, and maintenance of postsynaptic density ultrastructure. These findings are consistent with a gut-derived neuroimmune checkpoint mechanism, though causal directionality remains to be determined. The microbiota-dependent, tissue-selective PPAR modulation observed with EAP differs from that of direct receptor agonists, which may inform its development as a functional food ingredient targeting metabolic-cognitive comorbidities. Several limitations should be acknowledged. The male-only design and 14-week intervention window may limit generalizability to females and chronic disease models. The cross-sectional design permits association but not temporal precedence or causality. The relative contributions of central versus peripheral SCFA signaling and the necessity of specific SCFA species remain to be determined.

Future work should address these limitations through sex-comparative designs and longitudinal time-course analyses. Pathway-specific interventions—such as SCFA receptor antagonism, isotope-labeled metabolite tracing, and hepatocyte-specific PPAR knockout-will be needed to confirm the causal role of the proposed gut–liver–brain axis. Clarifying these mechanisms will be essential to translating the present preclinical findings into targeted dietary strategies for metabolic syndrome patients with early cognitive decline.

## Figures and Tables

**Table 1:** Sequences of primers for genes.

	Forward Primer	Reverse Primer
***GAPDH***	TGGAGAAACCTGCCAAGTATGA	TGGAAGAATGGGAGTTGCTGT
***IL-1β***	GCTACCTGTGTCTTTCCCGT	CGTCGACACACCAGCAGGTTA
***TNF-*** **α**	GCCAACGGCATGGATCTCAA	GATAGCAAATCGGCTGACGG
***Claudin-1***	TGAAGTGCATGAGGTGCCTG	CCACTAATGTCGCCAGACCTGA
***IL-6***	CTGCAAGAGACTTCCATCCAG	AGTGGTATAGACAGGTCTGTTGG

**Table 2:** Physiological indices and energy metabolism in HFFD-fed mice after 14-week EAP intervention.

**Physiological Index**	Control	HFFD	HFFD + LEAP	HFFD + HEAP
Food intake (g/mouse/day)	5.35 ± 0.45	4.29 ± 0.31 ^##^	4.83 ± 0.31	4.75 ± 0.42
Fluid intake (mL/mouse/day)	3.58 ± 0.35	4.08 ± 0.34 ^##^	3.9 ± 0.28	3.73 ± 0.29
Energy intake (kcal/mouse/day)	18.53 ± 2.64	22.74 ± 1.39 ^##^	20.56 ± 1.92 *	19.54 ± 1.56 **
FER	0.64 ± 0.07	0.95 ± 0.08 ^##^	0.73 ± 0.07 *	0.71 ± 0.06 **
TC (mmol/L)	2.5 ± 0.5	5.2 ± 0.4 ^##^	4.6 ± 0.6 *	3.1 ± 0.4 **
TG (mmol/L)	0.8 ± 0.1	1.5 ± 0.11 ^##^	1.1 ± 0.1 *	0.9 ± 0.09 **
LDL-C (mmol/L)	0.57 ± 0.09	1.46 ± 0.06 ^##^	1.29 ± 0.08 *	1.12 ± 0.07 **
HDL-C (mmol/L)	1.15 ± 0.06	0.63 ± 0.07 ^##^	0.74 ± 0.06 *	1.02 ± 0.05 **

Data are presented as mean ± SEM, n = 15. ^##^ *p* < 0.01 versus control group, * *p* < 0.05, ** *p* < 0.01 versus HFFD group.

## Data Availability

The original contributions presented in this study are included in the article/Supplementary Materials. Further inquiries can be directed to the corresponding author.

## Supplementary Materials

The following supporting information can be downloaded at https://www.mdpi.com/article/10.3390/foods15101794/s1, Figure S1: Impact of EAP on LEfSe-identified differentially abundant ASVs in HFFD-induced obese mice. (A) LDA scores of ASVs enriched in Control vs HFFD; (B) LDA scores of ASVs enriched in HFFD + EAP vs HFFD; Figure S2: Effects of EAP on top 50 genera in HFFD-induced obese mice. Heatmap of top 50 genera.

## Abbreviations

The following abbreviations are used in this manuscript:

EAP	*Elaeagnus angustifolia* polysaccharide
HPLC	high-performance liquid chromatography
NORT	novel object recognition test
OFT	open field test
MWM	Morris water maze
H&E	hematoxylin and eosin
NF-κB	nuclear factor-κB
PSD-95	postsynaptic-density protein 95
IBA-1	ionized calcium-binding adapter molecule 1
IL-6	interleukin-6
TNF-α	tumor necrosis factor-α
LPS	lipopolysaccharide
