# Supplementary material for: Elaeagnus angustifolia L. Polysaccharide Alleviates High-Fat High-Fructose Diet (HFFD)-Induced Cognitive Impairment by Modulating the Gut-Liver-Brain Axis
Source: Foods. 2026 May 19;15(10):1794. doi: 10.3390/foods15101794 (PMC13205740; doi:10.3390/foods15101794)
Supplement: Supplementary file 1 [file foods-15-01794-s001.zip › foods-4250250-supplementary.pdf]

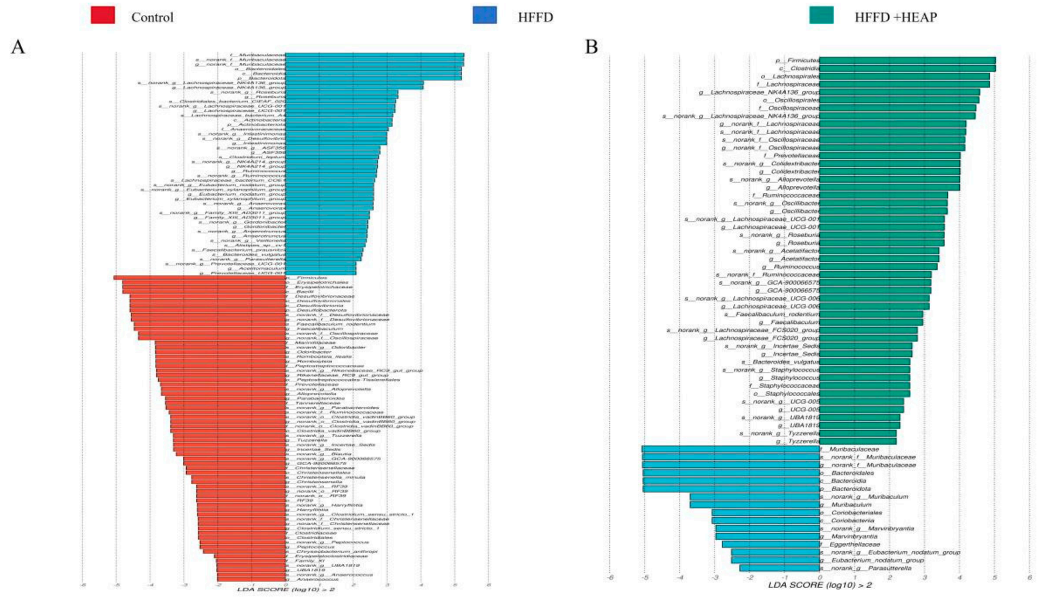

Fig S1 Supplementary Figure 1 Impact of EAP on LEfSe-identified differentially abundant ASVs in HFFD-induced obese mice

(A) LDA scores of ASVs enriched in Control vs HFFD; (B) LDA scores of ASVs enriched in HFFD + EAP vs HFFD. (Only ASVs with relative abundance ≥ 0.01, LDA score > 2 and P < 0.05 are shown) Data are expressed as the mean ± SEM, n=3. #p <0.05, ##p < 0.01 versus control group, *p < 0.05, **p < 0.01 versus HFFD group.

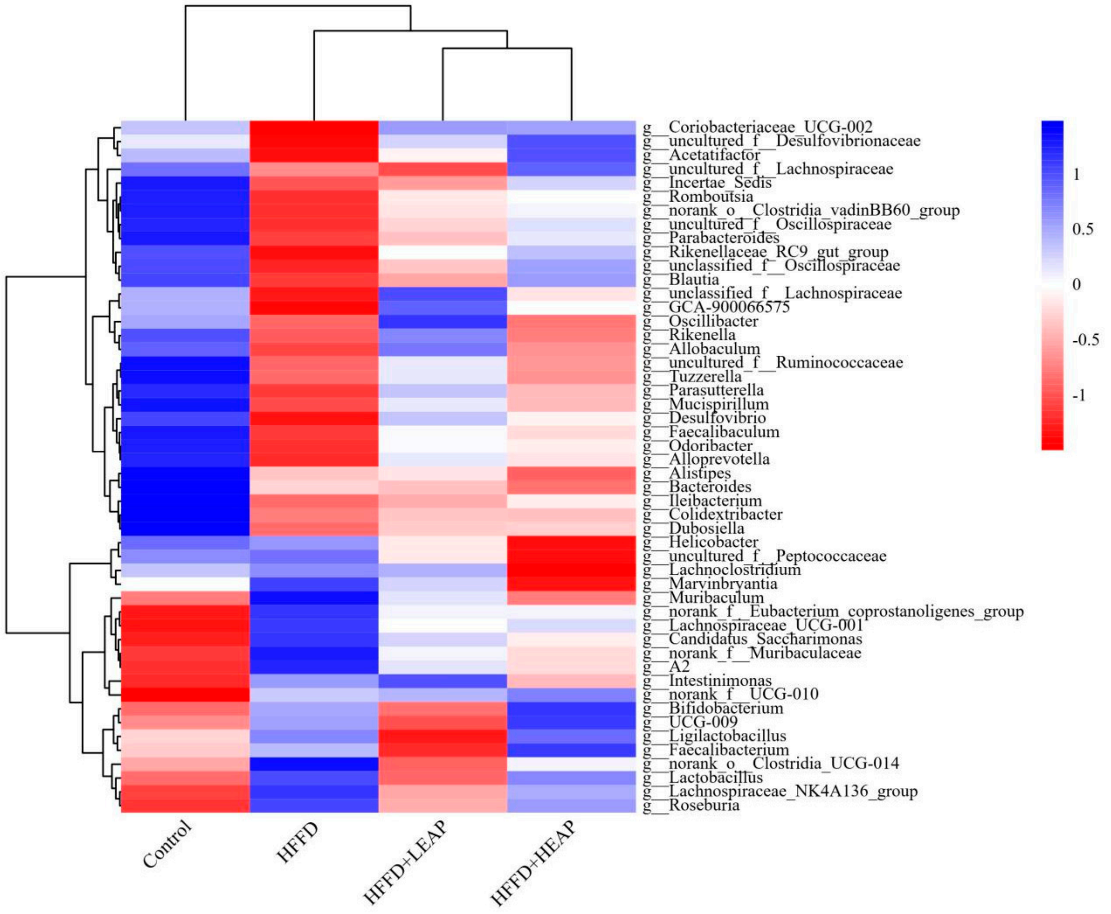

Supplementary Figure S2 Effects of EAP on top 50 genera in HFFD-induced obese mice

Heatmap of top 50 genera.
